# The Association Between Physical Activity and Frailty: China Health and Retirement Longitudinal Study (CHARLS)

**DOI:** 10.3390/ijerph22081219

**Published:** 2025-08-04

**Authors:** Wupeng Yin, Ximeng Zhao, Ayodele Tyndall, Nan Hu

**Affiliations:** 1 Department of Biostatistics, FIU Robert Stempel College of Public Health and Social Work, Miami, FL 33199, USA; 2 Department of Epidemiology, FIU Robert Stempel College of Public Health and Social Work, Miami, FL 33199, USA; xzhao023@fiu.edu; 3 Department of Dietetics and Nutrition, FIU Robert Stempel College of Public Health and Social Work, Miami, FL 33199, USA; 4 Department of Family and Preventive Medicine, University of Utah School of Medicine, Salt Lake City, UT 84132, USA

**Keywords:** physical activity, frailty, aging, longitudinal study, frailty index, China Health and Retirement Longitudinal Study

## Abstract

Background: With China’s rapidly aging population, frailty has become a growing concern among older adults. Physical activity (PA) is known to mitigate frailty-related decline, yet few studies have examined these associations longitudinally. Methods: Using five waves (2011–2020) of CHARLS data, we analyzed Chinese adults aged 60+ to assess the association between frailty—measured by a frailty index (FI)—and PA across various types (light, moderate, vigorous, total, and leisure). A generalized linear mixed-effects model was used, adjusting for demographic, socioeconomic, and health-related factors. Results: All PA types were significantly associated with lower odds of concurrent frailty, including light (OR = 0.37), moderate (OR = 0.37), vigorous (OR = 0.40), total (OR = 0.23), and leisure PA (OR = 0.56). Lagged PA also predicted reduced frailty risk over time, except for light PA. Conclusion: Regular PA is linked to a lower risk of frailty among older Chinese adults. These findings underscore the importance of sustained PA as a strategy to promote healthy aging and inform public health interventions for this population.

## 1. Introduction

Globally, the older adult population, above 65 years, is expected to reach approximately 1.5 billion by 2050 [1]. China is one of the countries with rapid growth in the aging population. In China, the number of individuals 60 years and over were 264 million in 2020, which accounted for 18.7% of the total population [2]. As the number of older adults increases, there is a growing concern for frailty, as it can affect the quality of life of older individuals [3,4]. It has been estimated that between a quarter and a half of older adults above 85 years are frail [4,5].

Frailty is a clinical state that increases the vulnerability of a person when they encounter a stressor because they have a decline in function and reserves for several physiologic systems [6,7,8]. Frailty leads to more dependency or mortality due to decreased capacity for resilience [5,7]. It is a risk factor for adverse health outcomes such as falls [9] and institutionalization [10] as well as poor outcomes after surgical procedures [11]. Frailty has been linked to higher use and cost of healthcare services [12]. The condition of frailty changes or increases with age [7,13].

Further, Wu et al. [14] highlighted in a study on the baseline China Health and Retirement Longitudinal Study (CHARLS) that 7% of older Chinese adults, 65 years or older, were frail, with higher rates of frailty in women and individuals with low levels of education [14]. The high rates of frailty in Chinese older adults and the rapid growth of the aging population in China will lead to greater healthcare needs. Studies on frailty based on the CHARLS found an increase in hospitalization and outpatient visits linked to frailty [15,16]. These studies identify the need to target potential modifiable factors that are major contributors to frailty such as physical inactivity. Every older adult is at risk of developing frailty as the aging population increases [17]. The increased risk of frailty in the older adult population as well as the increased use and cost of health services will lead to significant implications for public health. Therefore, it is essential to prevent, detect and manage frailty.

There are several validated measures to assess and diagnose frailty [4]. The two most common measures are the frailty index (FI) and the Fried frailty phenotype [18]. FI is considered a comprehensive validated measure to assess frailty because it considers several variables of health deficits which may increase the risk of frailty [4,19,20]. Thus, a greater deficit count will likely indicate an older adult who is more frail and more vulnerable to adverse health outcomes [19,21]. Therefore, early assessment and diagnosis of frailty for intervention has the potential to decrease healthcare cost, to reduce adverse health outcomes and to improve the quality of life for older adults at risk [4].

Physical activity (PA) is a modifiable approach to manage and reduce the risk of frailty. PA is categorized as any bodily movement that the skeletal system produces that increases the expenditure of energy [22]. PA has the potential to improve and preserve the function of many body systems. A form of PA is physical exercise, which improves and maintains components of physical fitness such as flexibility, balance, muscular strength, and cardio endurance [22]. An intervention with multiple components was proposed as the most effective option for frail individuals [23].

Moreover, effectiveness of a PA program may depend on the type, duration, frequency and intensity of exercises, and this tends to vary based on the individual. The American College of Cardiology/American Heart Association (ACC/AHA) suggest that regular PA is extremely beneficial to older adults [24]. ACC/AHA recommends regular PA including muscle strengthening and aerobic activity to promote healthy aging in older adults [24]. Also, the World Health Organization (WHO) highlights that PA improves functional ability, reduces bone health decline and prevents falls and associated injuries. WHO strongly recommends a minimum of 150–300 min of moderate-intensity aerobic PA, or a minimum of 75–150 min of vigorous-intensity aerobic activity weekly for older adults [25], as well as moderate intensity or greater muscle-strengthening activities for two or more days per week.

In addition, WHO recommends moderate or greater intensity of multicomponent PA emphasizing strength training and functional balance, for three or more days per week [25]. Thus, this recommendation aims to prevent falls and improve the functional capacities of older adults [25]; although, for frail adults these recommendations may require adaptations [22,25]. Several studies have shown that PA is beneficial and reduces the risk of frailty [17,26,27,28,29]. Although, few longitudinal studies based on the CHARLS examined frailty [7,14,29,30]. Further, to our current knowledge, only one longitudinal study specifically focused on exploring the relationship between varied levels of PA and frailty in Chinese adults using data from four waves of the CHARLS [29].

Given that frailty is an age-associated condition that contributes to early mortality and functional decline in older adults, early detection is crucial. More importantly, longitudinal studies on the effects of varied levels of PA are needed to guide effective implementation of PA interventions, and establish guidelines that will reduce the risk of frailty for older adults in China. This study uses the longitudinal data from five waves (2011–2020) of the CHARLS [31], to investigate the association between frailty and PA among late middle-age and older Chinese adults to explore the frailty status over time. The objective of this study is to examine the longitudinal association between different intensity levels and types of PA—including vigorous (VPA), moderate (MPA), and light (LPA) activity, as well as total physical activity (TPA) and leisure physical activity (LePA)—and frailty status among older Chinese adults. We hypothesize that engagement in different types of physical activity is associated with both concurrent and future frailty status.

## 2. Materials and Methods

### 2.1. Data Source and Study Population

This study utilized data from the China Health and Retirement Longitudinal Study (CHARLS), a nationally representative longitudinal survey designed to assess the health, economic status, and social well-being of individuals aged 45 years and older residing in mainland China. The baseline survey was conducted in 2011–2012, followed by follow-up waves in 2013 (Wave 2), 2015 (Wave 3), 2018 (Wave 4), and 2020 (Wave 5). CHARLS employed a stratified, multi-stage, probability proportional to size (PPS) sampling strategy, with stratification based on region, urban versus rural residence, and GDP per capita. The baseline sample covered 28 provinces, 150 counties or districts, and 450 villages or urban communities across China, and was weighted to align with the demographic distribution of the 2010 China Population Census, ensuring both national and regional representativeness [32]. Ethical approval for all waves of CHARLS was granted by the Institutional Review Board (IRB) at Peking University (IRB00001052-11015). The approval date was 17 January 2011 [33].

This longitudinal study utilized data from the China Health and Retirement Longitudinal Study (CHARLS), covering up to five waves of data collection (2011–2020). Two separate longitudinal datasets were constructed to accommodate differences in the availability of PA information across waves. The first dataset included data from all five waves and contained information on light, moderate, vigorous, and total PA. This dataset was used to examine the association between the FI and these specific types of PA (*n* = 25,873). The second dataset included only Waves 2 to 5 (*n* = 24,885), as these were the waves in which type-specific PA—particularly leisure-time PA—was collected. This restricted dataset was used to assess the association between FI and leisure physical activity.

For each dataset, the earliest wave in which a participant was observed (Wave 1 for the full dataset, or Wave 2 for the restricted dataset) was designated as that participant’s baseline, with subsequent waves serving as follow-up assessments. Participants were not required to enter the study in the same wave, and individuals could contribute data from any wave in which they participated. Thus, the baseline for participants was time of their first available measurement in the dataset.

To construct the analytic cohorts, participants from each dataset were included if they were aged 60 years or older and had at least two non-missing FI measurements. In the dataset containing light (LPA), moderate (MPA), vigorous (VPA), and total physical activity (TPA), 17,151 individuals were excluded due to missing age information or being under age 60, and 1221 were excluded for having FI data from only one wave, resulting in a final cohort of 7501 individuals. In the restricted dataset containing leisure physical activity (LePA), 16,758 individuals were excluded due to age-related criteria, and 1328 were excluded for having only one FI measurement, yielding a final sample of 6799 individuals.

### 2.2. Measurements

#### 2.2.1. Measurement of FI

Drawing Frailty was measured using the FI, which quantifies the accumulation of age-related health deficits. The FI was constructed following standardized procedures established in the literature [20]. Drawing on prior studies that utilized the CHARLS dataset to derive FI scores [34,35,36], we developed the FI using 35 variables across five types: chronic conditions and symptoms, functional disabilities, mobility limitations, depressive symptoms, and cognitive function. These variables were extracted from five survey waves (2011, 2013, 2015, 2018, and 2020) in the China Health and Retirement Longitudinal Study (CHARLS) (see Appendix A, Table A1).

Each of the 35 variables was dichotomized according to established criteria, where 0 indicated no deficit and 1 represented the presence of a full deficit. For Waves 1–4, the FI was calculated by dividing the total number of observed deficits by the number of non-missing items, provided that the proportion of missing variables did not exceed 15% (i.e., more than 5 missing items). If a participant had more than 15% missing data, the FI was considered missing. In Wave 5, due to the absence of mobility-related data, the FI was constructed from 28 variables. Accordingly, if more than 4 items were missing, the FI value was treated as missing. The resulting FI scores ranged continuously from 0 to 1, with higher values reflecting greater frailty.

Consistent with prior research [36,37], the FI was further dichotomized for categorical analyses: individuals with FI scores below 0.25 were classified as non-frail, while those with scores of 0.25 or higher were classified as frail.

#### 2.2.2. Assessment of PA

PA was assessed using questionnaire items from CHARLS, which adopted a format consistent with the short form of the International Physical Activity Questionnaire (IPAQ) [38]. Participants were asked whether they engaged in PA for at least 10 consecutive minutes during a typical week at three intensity levels: VPA, including activities such as heavy lifting, digging, aerobic exercise, or fast cycling; MPA, including tasks like carrying light loads, Tai Chi, or brisk walking; and LPA, such as walking for transportation, leisure, or light household duties. For each reported activity, follow-up questions captured the number of days per week (1–7), duration (categorized as 10–30 min, 30 min–2 h, 2–4 h, or more than 4 h), and the purpose of the activity (e.g., leisure or exercise).

VPA, MPA, and LPA were defined based on participants engaging in the respective activity for a minimum of 10 consecutive minutes during a usual week. Participants who did not meet this criterion were classified as physically inactive at that activity level.

To evaluate the cumulative effects of physical activity, two summary indicators were created. TPA combined VPA, MPA, and LPA across all types, while LePA included only activities performed for leisure or exercise. For each intensity level, weekly duration was estimated using the midpoint of each time category, with durations exceeding 4 h capped at 4 h. Physical activity volume scores were calculated by applying metabolic equivalent (MET) [39,40,41] values as follows:

(1) Total Physical Activity Volume (TPAV) = 8.0 × total VPA duration + 4.0 × total MPA duration + 3.3 × total LPA duration;
(2) Leisure Physical Activity Volume (LePAV) = 8.0 × leisure VPA duration + 4.0 × leisure MPA duration + 3.3 × leisure LPA duration.

Following IPAQ guidelines, individuals who accumulated at least 600 MET-minutes per week for TPAV or LePAV were considered physically active. Those who did not meet this threshold were categorized as physically inactive.

#### 2.2.3. Covariates

Covariates were selected based on the prior literature [18,25,28,29] and the availability of relevant data in CHARLS, with consideration given to their potential role as confounders of the primary association. The following variables were included: time-period effect (in years), baseline age (in years), gender (male = 0, and female = 1), education level (less than lower secondary = 0, upper secondary and vocational training = 1, tertiary = 2), BMI (underweight = 0, normal weight = 1, overweight = 2, obesity = 3), marital status (not married = 0, married = 1), residency (urban = 0, rural = 1), income (no income = 0, any income = 1), drinking (non-drinker = 0, current drinker = 1), and smoking (non-smoker = 0, current smoker = 1). Body mass index (BMI) was categorized into four groups based on the World Health Organization’s guidelines for Asian and South Asian populations [42]: underweight (≤18.5 kg/m^2^), normal weight (18.5–23.9 kg/m^2^), overweight (24.0–24.9 kg/m^2^), and obese (≥25.0 kg/m^2^). Income was categorized based on whether the respondent reported receiving any wages or bonus income in the past year. For alcohol consumption, a code of 0 indicates that the respondent did not report consuming any alcoholic beverages in the past 12 months. For smoking status, a code of 0 indicates that the respondent had previously smoked but had quit at the time of the survey.

### 2.3. Statistical Analysis

Descriptive statistics were used to summarize the characteristics of the study population. Categorical variables were presented as frequency (N) and percentage (%), while continuous variables were expressed as means and standard deviations (mean ± SD) for those that were normally or approximately normally distributed. To compare baseline characteristics between physically active and inactive groups across different PA types, chi-squared (χ^2^) tests were used for categorical variables, and two-sample Student’s *t*-tests were applied for continuous variables.

The given the nested and longitudinal structure of the CHARLS dataset—where repeated observations are recorded for individuals over time (Level 1), nested within households (Level 2), and further nested within communities (Level 3)—a Generalized Linear Mixed-Effects Model (GLMM) [43] was employed to account for the multilevel dependencies in the data. The outcome variable, frailty status, is binary in nature; therefore, the outcome was assumed with a binomial distribution and the GLMM uses a logit link function. To address within-subject correlation arising from repeated measurements, a random intercept was included at the individual level. Additional random intercepts at the household and community levels were incorporated to account for higher-order clustering effects. This modeling framework improves the precision of fixed-effect estimates by adjusting for unobserved heterogeneity across these nested levels [41,44].

The GLMMs were fitted using maximum likelihood estimation with bound optimization via quadratic approximation. For each model, the odds ratio (OR), 95% confidence interval (CI), and *p*-value for the fixed effects were reported. Analyses were conducted separately for each outcome-exposure pair, with one dependent and one independent variable included per model specification:

(1) $$g\left(E\left(y_{ijkt}|u_{i},u_{j\left(i\right)},u_{k\left(j\left(i\right)\right)}\right)\right)=X_{ijkt}\beta +u_{i}+u_{j\left(i\right)}+u_{k(j(i))}+e_{ijkt}$$

In the model formulation, $y_{ijkt}$ denotes the outcome for individual k in household j, within community i, at time t. The design matrix of fixed effects is represented by $X_{ijkt}$, which includes the exposure variable, time since baseline, and other covariates used for adjustment. The corresponding vector of fixed-effect coefficients is denoted by β. Random intercepts were included at multiple levels to account for hierarchical structure: $u_{i}\;\sim \;N(0,\;\sigma_{h}^{2})$ represents individual-level variation capturing within-subject correlation from repeated measures; $u_{j(i)}\;\sim \;N(0,\;\sigma_{h}^{2})$ denotes household-level random effects to account for shared environmental or contextual influences; and $u_{k(j(i))}\;\sim \;N\left(0,\;\sigma_{c}^{2}\right)$ represents community-level random effects capturing broader reginal variation. The residual error is denoted by $e_{ijkt}\;\sim \;N(0,\;\sigma_{e}^{2})$. For binary outcomes, a logistic link function was applied, defined as $g\left(x\right)=ln(\frac{x}{1-x})$.

All statistical analyses were conducted using R software (version 4.4.2; cran.r-project.org) and SAS (SAS Institute Inc., Cary, NC, USA) version 9.4. Statistical significance was determined with two-sided tests, with a *p*-value threshold of <0.05. Missing data for key covariates such as BMI, income, smoking, and drinking were addressed using complete case analysis. This approach was implemented automatically through the lme4 package in R, which excludes participants with missing values in any model-specific covariates. While this method ensures consistency across model estimates, it may reduce sample size and introduce selection bias. We have acknowledged this limitation in Section 4

## 3. Results

### 3.1. Descriptive Statistics

Table 1 presents the baseline characteristics of 7501 older adults aged 60 and above included in the full longitudinal dataset (Waves 1–5), stratified by PA types: LPA, MPA, VPA, and TPA. The overall prevalence of frailty at baseline (Wave 1) was 19.16%, with variations across PA subgroups (Table 1). The mean age at baseline was 67.90 ± 5.65 years, and 50.1% were male. Higher levels of PA engagement were significantly associated with younger age across all types (*p* < 0.001). Males were more likely to engage in MPA, VPA, and TPA (*p* < 0.001), whereas no gender difference was observed for LPA.

Participants with higher educational attainment, particularly those with upper secondary or tertiary education, were more likely to engage in moderate, vigorous, and total PA (*p* < 0.001). Significant associations were also observed between PA and BMI, marital status, residency, income, drinking, smoking status, and frailty status. Notably, individuals classified as frail were less likely to engage in any type of PA (all *p* < 0.001).

Table 2 shows the characteristics of 6799 participants from the restricted dataset (Waves 2–5), used to assess the association between frailty and LePA. The mean age was 67.51 ± 6.25 years, and 49.24% were male. Leisure PA engagement was significantly associated with younger age (*p* < 0.001), higher education (*p* < 0.001), lower BMI (*p* < 0.001), being married (*p* < 0.001), urban residency (*p* < 0.001), any income (*p* < 0.001), and non-smoking and non-drinking status (*p* < 0.001 for both).

Importantly, the prevalence of frailty (FI ≥ 0.25) was significantly lower among participants reporting leisure PA (18.54%) compared to those not reporting leisure PA (22.89%) (*p* < 0.001), suggesting a potential protective role of LePA in mitigating frailty risk.

### 3.2. Association Between FI and Concurrent PA Among Older Chinese Adults

Table 3 presents the associations between FI and concurrent engagement in four PA types—LPA, MPA, VPA, and TPA—using longitudinal data from Waves 1 to 5 of CHARLS. Results from both unadjusted and adjusted generalized linear mixed-effects models are shown.

In the unadjusted models, engagement in any type of PA was significantly associated with lower odds of frailty. Compared to participants who did not engage in PA, those who participated in LPA, MPA, and VPA had 63% (OR = 0.37, 95% CI: 0.33–0.42), 63% (OR = 0.37, 95% CI: 0.33–0.41), and 60% (OR = 0.40, 95% CI: 0.35–0.46) lower odds of being frail, respectively (all *p* < 0.001). Participants engaging in TPA had the greatest reduction in odds of frailty (OR = 0.23, 95% CI: 0.21–0.26, *p* < 0.001).

In the model adjusted for follow-up time, baseline age, gender, education level, BMI, marital status, residency, income, drinking status, and smoking status, these associations remained statistically significant though slightly attenuated. The odds of frailty were 53% lower among those engaging in LPA (OR = 0.47, 95% CI: 0.41–0.55), 55% lower for MPA (OR = 0.45, 95% CI: 0.39–0.51), and 45% lower for VPA (OR = 0.55, 95% CI: 0.47–0.64). TPA remained strongly protective, with a 71% reduction in odds (OR = 0.29, 95% CI: 0.25–0.34). All associations were statistically significant (*p* < 0.001).

Table 4 reports the results for the association between FI and concurrent engagement in LePA, using the restricted dataset from Waves 2 to 5. In the unadjusted model, engaging in LePA was associated with 44% lower odds of frailty (OR = 0.56, 95% CI: 0.50–0.64, *p* < 0.001). This association remained significant after adjusting for the same covariates listed above, with participants who engaged in LePA showing 41% lower odds of frailty (OR = 0.59, 95% CI: 0.51–0.68, *p* < 0.001).

These findings provide consistent evidence that concurrent engagement in various types of PA—including light, moderate, vigorous, total, and leisure-time activity—is associated with a significantly lower likelihood of frailty among older Chinese adults.

### 3.3. Association Between FI and PA at Lagged and Current Time Points Among Older Chinese Adults

Table 5 and Table 6 present the associations between FI and engagement in PA across various types—LPA, MPA, VPA, TPA, and LePA—at both the current and lagged time points (i.e., one wave prior). Odds ratios (ORs), 95% confidence intervals (CIs), and *p*-values are reported for both unadjusted and adjusted models. The adjusted models accounted for follow-up time, baseline age, gender, education level, BMI, marital status, residency, income, drinking status, and smoking status.

In the unadjusted models, engagement in PA of all types—both current and lagged—was significantly associated with lower odds of frailty (all *p* < 0.001). For example, participants reporting current LPA had 64% lower odds of frailty (OR = 0.36, 95% CI: 0.31–0.42), while those reporting lagged LPA had 42% lower odds (OR = 0.58, 95% CI: 0.49–0.69). Similarly strong associations were observed for MPA, VPA, and TPA, with ORs ranging from 0.24 to 0.41. For LePA, unadjusted estimates indicated that current engagement was associated with 44% lower odds of frailty (OR = 0.56, 95% CI: 0.46–0.67), and lagged LePA with 25% lower odds (OR = 0.75, 95% CI: 0.62–0.90).

In the adjusted models, concurrent engagement in PA remained robustly associated with lower frailty risk. Specifically, current LPA (OR = 0.43, 95% CI: 0.35–0.51), MPA (OR = 0.43, 95% CI: 0.36–0.52), VPA (OR = 0.49, 95% CI: 0.39–0.60), and TPA (OR = 0.27, 95% CI: 0.22–0.33) were all significantly protective (*p* < 0.001). Lagged PA also demonstrated significant associations, though the effect sizes were generally attenuated. For example, lagged LPA (OR = 0.73, 95% CI: 0.60–0.88), MPA (OR = 0.71, 95% CI: 0.59–0.86), VPA (OR = 0.78, 95% CI: 0.64–0.96), and TPA (OR = 0.61, 95% CI: 0.50–0.75) remained significantly associated with lower frailty (all *p* < 0.05). In the restricted dataset (Table 6), after adjustment, current LePA showed an OR of 0.61 (95% CI: 0.51–0.74, *p* < 0.001), while lagged LePA had an OR of 0.77 (95% CI: 0.63–0.93, *p* = 0.007), suggesting a sustained protective effect over time.

These results underscore that both current and prior participation in PA is significantly associated with lower frailty among older Chinese adults, with concurrent PA showing slightly stronger effects.

## 4. Discussion

This longitudinal study provides comprehensive insights into the associations between frailty status and various types of PA among older adults in China, using up to five waves (2011–2020) of the CHARLS. By constructing two separate datasets to accommodate variations in PA measurements across waves, we examined the concurrent and one-wave lagged effects of LPA, MPA, VPA, TPA, and LePA on frailty, as measured by the FI. Our findings indicate that active engagement in all types of PA, including LPA, MPA, VPA, TPA, and LePA, is significantly associated with a lower likelihood of being frail at the same time point. This is consistent with the existing literature that demonstrates the protective role of PA against frailty by maintaining physical, functional, and metabolic health [22,23,24]. Prior cross-sectional and longitudinal studies have reported similar findings, particularly about moderate and vigorous PA, which have been shown to enhance muscular strength, mobility, and overall physiological resilience in aging populations [18,25,28].

We also examined the temporal association between frailty and PA by including PA exposures lagged by one wave (i.e., measured in the previous wave) and found that past engagement in MPA, VPA, TPA, and LePA was significantly associated with lower odds of being frail in the subsequent wave. These findings suggest that sustained physical activity is associated with a lower risk of frailty over time, underscoring the potential importance of maintaining regular activity in older adulthood [26,27,28].

Our findings align with and extend previous research conducted using the CHARLS dataset. For example, Wu et al. [14] reported baseline frailty prevalence patterns and demographic risk factors in older Chinese adults, providing a foundational understanding of frailty burden in this population. Ma et al. [30] further demonstrated inverse associations between PA and frailty using four waves of CHARLS, focusing primarily on intensity-specific PA. Our study builds on this work by incorporating five waves of data, examining both concurrent and lagged associations, and including domain-specific PA types such as leisure-time activity. These methodological extensions enhance the temporal and contextual insights into PA’s role in frailty prevention. Furthermore, our findings are consistent with recommendations from the Asian Working Group for Sarcopenia and Frailty [45], which emphasize PA as a modifiable factor for healthy aging in Asian populations.

To further explore the directionality of the relationship between frailty and physical activity, we conducted additional analyses in which the FI and its lagged value (one wave prior) were treated as the exposures and different PA types as outcomes. The results indicated that higher frailty levels were significantly associated with lower engagement in all types of PA at both current and subsequent time points. These findings suggest a potential bidirectional relationship in which frailty status may influence future PA behaviors. While our primary hypothesis—supported by the Bradford Hill criteria [46]—posits that engagement in PA is likely a protective factor against the development of frailty, this interpretation must be made cautiously. Future studies employing more robust causal inference methods, such as instrumental variable approaches or longitudinal mediation models, are needed to elucidate further the directionality and mechanisms linking PA and frailty. Moreover, it is plausible that frailty itself reduces individuals’ physical functioning, motivation, or capacity to remain active. This reciprocal association has been noted in previous research and highlights the complex interplay between health status and behavior. Future studies should apply robust causal inference techniques, such as instrumental variable methods, longitudinal mediation models, cross-lagged panel designs, or structural equation modeling, to better disentangle the temporal and directional nature of the relationship and to inform optimal intervention strategies.

While our use of one-wave lag models provided preliminary insight into temporal ordering, we recognize that this approach may not fully capture the delayed or cumulative biological effects of physical activity on frailty. Given that physiological adaptations often require sustained behavioral change and time to manifest, future research should explore multi-lag or cumulative exposure models to more comprehensively assess the long-term impact of physical activity on frailty progression.

From a public health perspective, our findings support existing recommendations for encouraging older adults to engage in regular physical activity, particularly activities of moderate or greater intensity and those performed in leisure contexts [47,48]. Such efforts may delay the onset of frailty and reduce the burden on healthcare systems. Our results also provide evidence to support tailored interventions [49,50] based on activity type and demographic characteristics. Beyond physiological effects, leisure PA may offer unique psychological and social benefits that contribute to frailty reduction. For example, LePA can enhance mental well-being, reduce stress and depressive symptoms, and promote social engagement—factors that have been independently linked to healthier aging outcomes. These multidimensional benefits underscore the need for future research to explore the psychosocial pathways through which LePA influences frailty, particularly in aging populations where isolation and cognitive decline are prevalent risks. Furthermore, the LePA analysis was limited to data from Waves 2–5, reducing the sample size and possibly attenuating effect estimates. These considerations should be explored in future studies that differentiate between PA domains with more precision and longer follow-up. However, translating these findings into practice requires attention to contextual barriers faced by older Chinese adults. For example, urban–rural disparities may impact access to exercise facilities, walkable environments, and health promotion resources. Cultural beliefs that view aging as a period of rest rather than activity may further limit motivation. Public health strategies should therefore incorporate culturally sensitive education campaigns, community-based exercise programs, and environmental modifications to create age-friendly spaces that encourage movement. Partnerships between local governments, community organizations, and healthcare providers can be instrumental in designing and implementing scalable PA interventions across different settings in China.

However, several limitations should be acknowledged. First, the classification and quantification of physical activity (PA) relied on self-reported data, which may be subject to recall bias, social desirability bias, and misclassification. These concerns are especially relevant in older populations, where cognitive impairments or subjective interpretations may lead to inaccuracies in reporting activity frequency, duration, or intensity. This measurement error is particularly relevant in older populations, where physical activity recall may be imprecise due to cognitive limitations or misinterpretation of intensity levels. Consequently, the observed associations—particularly the protective effects—may be attenuated, potentially underestimating the true impact of physical activity on frailty. For instance, participants may have misclassified light, moderate, and vigorous activities based on personal perception rather than objective metabolic equivalents. Such measurement error could attenuate the observed associations—particularly the protective effects—and obscure differences across PA intensity categories.

Second, although we adjusted for a wide range of sociodemographic and health-related variables—such as dietary intake, cognitive function, social support, and healthcare access—they may still have influenced the observed associations. Future research should incorporate more comprehensive covariate data and objective PA assessments (e.g., accelerometers) to improve measurement accuracy and internal validity.

Third, missing data in both the outcome and key covariates present additional limitations. In Wave 5, only 28 frailty index (FI) variables were available due to the absence of mobility-related data, compared to 35 variables in Waves 1–4. Additionally, complete case analysis was used to handle missing values for key covariates such as BMI and income. While these approaches ensured consistency across model estimates, they may have introduced bias by altering the measurement of frailty or reducing the effective sample size—particularly if data were not missing completely at random. To evaluate the robustness of our findings, we conducted sensitivity analyses, including a one-wave carry-forward approach using FI data from Wave 4 and a complete-case analysis subset. In both cases, the results were consistent with our primary findings, supporting the reliability of our conclusions. Nonetheless, the potential for selection bias remains, and future studies should consider applying advanced imputation methods and leveraging more complete data sources where feasible.

Fourth, because our study focused specifically on individuals aged 60 and above, a substantial number of participants from the initial CHARLS sample were excluded. While this age restriction aligns with our research objective of examining frailty among older adults, it may introduce selection bias if excluded individuals differed systematically in sociodemographic or health-related characteristics.

Fifth, although our analytical approach incorporated lagged PA to approximate temporal direction, causal inference cannot be established. Furthermore, due to the absence of type-specific PA data in Wave 1, we constructed a restricted dataset beginning from Wave 2 to assess domain-specific associations (e.g., leisure PA). While this allowed us to examine differences by PA type, it may have introduced selection bias and reduced comparability across domains, thereby limiting analytical symmetry and interpretability.

Lastly, while we did not include interaction or subgroup analyses in the main text, we conducted a subgroup analysis by gender and found consistent results across groups, further supporting the robustness of our primary findings. However, these results were not included in the manuscript, as the goal of this study was not to examine disparities or population-specific effect modification, but rather to assess the overall longitudinal association between physical activity and frailty. Future research should explore potential subgroup differences (e.g., by region or education) and consider alternative FI thresholds (e.g., 0.21 or 0.30) to better inform targeted and equitable public health strategies.

Despite these limitations, our study contributes valuable longitudinal evidence that highlights the importance of type-specific and sustained PA for frailty prevention among older Chinese adults. Continued efforts to promote regular PA across multiple types, especially in aging populations, are critical for healthy aging and resilience.

## 5. Conclusions

Despite its limitations, this longitudinal study contributes valuable evidence on the association between different types of physical activity and frailty prevention among older Chinese adults. While our findings demonstrate that regular engagement in physical activity—particularly of moderate or greater intensity—is associated with a lower likelihood of being frail over time, these associations should not be interpreted causally given the observational nature of the study. The results must be considered in light of several limitations, including the high rate of missing data in key covariates, the potential for reverse causation, and the use of self-reported physical activity measures that may introduce bias. Furthermore, we observed differences in the strength of association between total and leisure-time physical activity, underscoring the need to interpret activity types separately. Future research should incorporate more robust causal inference methods, objective assessments of physical activity, and strategies to address missing data, while also considering the practical challenges and policy implications for promoting physical activity among diverse aging populations in China.

## Figures and Tables

**Table 1:** Characteristics of participants in study cohort (Waves 1–5, *n* = 7501).

Variables		All Subjects (*n* = 7501)	Light Physical Activity (LPA) (*n* = 2647)			Moderate Physical Activity (MPA) (*n* = 2657)			Vigorous Physical Activity (VPA) (*n* = 2657)			Total Physical Activity (TPA) (*n* = 2627)		
			No *n* = 527	Yes *n* = 2120	*p Value*	No *n* = 1330	Yes *n* = 1327	*p Value*	No *n* = 1932	Yes *n* = 725	*p Value*	No *n* = 580	Yes *n* = 2047	*p Value*
Age at baseline (years), Mean (SD)		67.90 (6.55)	68.99 (7.33)	67.43 (6.12)	<0.001	68.99 (6.92)	66.49 (5.56)	<0.001	68.43 (6.69)	65.89 (5.12)	<0.001	70.07 (7.36)	67.09 (5.95)	<0.001
Gender, *n* (%)	Male	3760 (50.13)	232 (44.02)	1071 (50.52)	0.015	614 (46.17)	691 (52.07)	0.004	860 (44.51)	445 (61.38)	<0.001	234 (40.34)	1062 (51.88)	<0.001
	Female	3741 (49.87)	295 (55.98)	1049 (49.48)		716 (53.83)	636 (47.93)		1072 (55.49)	280 (38.62)		346 (59.66)	985 (48.12)	
Education level, *n* (%)	Less than lower secondary	6964 (92.84)	512 (97.15)	1959 (92.41)	0.002	1239 (93.16)	1242 (93.59)	0.588	1778 (92.03)	703 (96.97)	<0.001	559 (96.38)	1892 (92.43)	0.007
	Upper secondary and vocational training	383 (5.11)	12 (2.28)	109 (5.14)		62 (4.66)	59 (4.45)		105 (5.43)	16 (2.21)		17 (2.93)	104 (5.08)	
	Tertiary	154 (2.05)	3 (0.57)	52 (2.45)		29 (2.18)	26 (1.96)		49 (2.54)	6 (0.83)		4 (0.69)	51 (2.49)	
BMI, *n* (%)	Underweight	556 (7.41)	51 (9.68)	173 (8.16)	<0.001	106 (7.97)	118 (8.89)	<0.001	147 (7.61)	77 (10.62)	<0.001	58 (10.00)	163 (7.96)	<0.001
	Normal weight	2401 (32.01)	167 (31.69)	831 (39.20)		454 (34.14)	550 (41.45)		651 (33.70)	353 (48.69)		184 (31.72)	806 (39.37)	
	Overweight	1022 (13.62)	79 (14.99)	332 (15.66)		196 (14.74)	215 (16.20)		312 (16.15)	99 (13.66)		81 (13.97)	326 (15.93)	
	Obesity	1416 (18.88)	124 (23.53)	479 (22.59)		319 (23.98)	286 (21.55)		482 (24.95)	123 (16.97)		137 (23.62)	462 (22.57)	
	Missing	2106 (28.08)	106 (20.11)	305 (14.39)		255 (19.17)	158 (11.91)		340 (17.60)	73 (10.07)		120 (20.69)	290 (14.17)	
Marital status, *n* (%)	Not married	1412 (18.82)	113 (21.44)	441 (20.80)	<0.001	333 (25.04)	222 (16.73)	<0.001	449 (23.24)	106 (14.62)	<0.001	156 (26.90)	392 (19.15)	<0.001
	Married	5224 (69.64)	414 (78.56)	1679 (79.20)		997 (74.96)	1105 (83.27)		1483 (76.76)	619 (85.38)		424 (73.10)	1655 (80.85)	
	Missing	865 (11.53)	0 (0.00)	0 (0.00)		0 (0.00)	0 (0.00)		0 (0.00)	0 (0.00)		0 (0.00)	0 (0.00)	
Residency, *n* (%)	Urban	3049 (40.65)	194 (36.81)	827 (39.01)	0.017	588 (44.21)	434 (32.71)	<0.001	860 (44.51)	162 (22.34)	<0.001	238 (41.03)	779 (38.06)	0.019
	Rural	4452 (59.35)	333 (63.19)	1293 (60.99)		742 (55.79)	893 (67.29)		1072 (55.49)	563 (77.66)		342 (58.97)	1268 (61.94)	
Income, *n* (%)	No income	6002 (80.02)	490 (92.98)	1929 (90.99)	<0.001	1248 (93.83)	1180 (88.92)	<0.001	1808 (93.58)	620 (85.52)	<0.001	558 (96.21)	1845 (90.13)	<0.001
	Any income	610 (8.13)	36 (6.83)	184 (8.68)		78 (5.86)	143 (10.78)		116 (6.00)	105 (14.48)		21 (3.62)	196 (9.57)	
	Missing	889 (11.85)	1 (0.19)	7 (0.33)		4 (0.30)	4 (0.30)		8 (0.42)	0 (0.00)		1 (0.17)	6 (0.29)	
Drinking, *n* (%)	Non-drinker	4621 (61.61)	399 (75.71)	1476 (69.62)	<0.001	996 (74.89)	886 (66.77)	<0.001	1432 (74.12)	450 (62.07)	<0.001	451 (77.76)	1408 (68.78)	<0.001
	Current drinker	2003 (26.70)	128 (24.29)	644 (30.38)		334 (25.11)	441 (33.23)		500 (25.88)	275 (37.93)		129 (22.24)	639 (31.22)	
	Missing	877 (11.69)	0 (0.00)	0 (0.00)		0 (0.00)	0 (0.00)		0 (0.00)	0 (0.00)		0 (0.00)	0 (0.00)	
Smoking, *n* (%)	Non-smoker	4579 (61.05)	387 (73.43)	1491 (70.33)	<0.001	975 (73.31)	910 (68.58)	<0.001	1440 (74.53)	445 (61.38)	<0.001	438 (75.52)	1426 (69.66)	<0.001
	Current smoker	1889 (25.18)	140 (26.57)	629 (29.67)		355 (26.69)	417 (31.42)		492 (25.47)	280 (38.62)		142 (24.48)	621 (30.34)	
	Missing	1033 (13.77)	0 (0.00)	0 (0.00)		0 (0.00)	0 (0.00)		0 (0.00)	0 (0.00)		0 (0.00)	0 (0.00)	
FI, *n* (%)	Non-frail	5123 (68.30)	345 (65.46)	1698 (80.09)	<0.001	951 (71.50)	1101 (82.97)	<0.001	1443 (74.69)	609 (84.00)	<0.001	354 (61.03)	1672 (81.68)	<0.001
	Frail	1437 (19.16)	181 (34.35)	401 (18.92)		370 (27.82)	213 (16.05)		480 (24.84)	103 (14.21)		225 (38.79)	355 (17.34)	
	Missing	941 (12.54)	1 (0.19)	21 (0.99)		9 (0.68)	13 (0.98)		9 (0.47)	13 (1.79)		1 (0.17)	20 (0.98)	

**Table 2:** Characteristics of participants in study cohort (Waves 2–5, *n* = 6799).

Variables		All Subjects (*n* = 6799)	By Leisure Physical Activity (LePA) Status (*n* = 2067)		
			No *n* = 1490	Yes *n* = 577	*p Value*
Age at baseline (years), Mean (SD)		67.51 (6.25)	67.00 (5.98)	67.74 (6.08)	<0.001
Gender, *n* (%)	Male	3383 (49.76)	720 (48.32)	312 (54.07)	0.063
	Female	3416 (50.24)	770 (51.68)	265 (45.93)	
Education level, *n* (%)	Less than lower secondary	6335 (93.18)	1434 (96.24)	507 (87.87)	<0.001
	Upper secondary and vocational training	343 (5.04)	49 (3.29)	44 (7.63)	
	Tertiary	121 (1.78)	7 (0.47)	26 (4.51)	
BMI, *n* (%)	Underweight	425 (6.25)	106 (7.11)	22 (3.81)	<0.001
	Normal weight	2004 (29.47)	549 (36.85)	137 (23.74)	
	Overweight	954 (14.03)	221 (14.83)	108 (18.72)	
	Obesity	1384 (20.36)	289 (19.40)	178 (30.85)	
	Missing	2032 (29.89)	325 (21.81)	132 (22.88)	
Marital status, *n* (%)	Not married	1439 (21.16)	327 (21.95)	120 (20.80)	<0.001
	Married	4882 (71.80)	1162 (77.99)	456 (79.03)	
	Missing	478 (7.03)	1 (0.07)	1 (0.17)	
Residency, *n* (%)	Urban	2691 (39.58)	405 (27.18)	332 (57.54)	<0.001
	Rural	4108 (60.42)	1085 (72.82)	245 (42.46)	
Income, *n* (%)	No income	5588 (82.19)	1300 (87.25)	520 (90.12)	<0.001
	Any income	681 (10.02)	186 (12.48)	57 (9.88)	
	Missing	530 (7.80)	4 (0.27)	0 (0.00)	
Drinking, *n* (%)	Non-drinker	4347 (63.94)	1050 (70.47)	397 (68.80)	<0.001
	Current drinker	1942 (28.56)	439 (29.46)	180 (31.20)	
	Missing	510 (7.50)	1 (0.07)	0 (0.00)	
Smoking, *n* (%)	Non-smoker	3997 (58.79)	943 (63.29)	399 (69.15)	<0.001
	Current smoker	865 (12.72)	244 (16.38)	68 (11.79)	
	Missing	1937 (28.49)	303 (20.34)	110 (19.06)	
FI, *n* (%)	Non-frail	4861 (71.50)	1146 (76.91)	469 (81.28)	<0.001
	Frail	1347 (19.81)	341 (22.89)	107 (18.54)	
	Missing	591 (8.69)	3 (0.20)	1 (0.17)	

**Table 3:** Association between FI and concurrent engagement in different types of PA based on longitudinal data from Waves 1 to 5 of CHARLS with results shown for both unadjusted and adjusted models.

Type of PA	FI					
	Unadjusted Model			Adjusted Model ^1^		
	*N*	OR (95% CI)	*p Value*	*N*	OR (95% CI)	*p Value*
Light Physical Activity (LPA)	6805			5716		
No		Ref			Ref	
Yes		0.37 (0.33, 0.42)	<0.001		0.47 (0.41, 0.55)	<0.001
Moderate Physical Activity (MPA)	6805			5720		
No		Ref			Ref	
Yes		0.37 (0.33, 0.41)	<0.001		0.45 (0.39, 0.51)	<0.001
Vigorous Physical Activity (VPA)	6804			5720		
No		Ref			Ref	
Yes		0.40 (0.35, 0.46)	<0.001		0.55 (0.47, 0.64)	<0.001
Total Physical Activity (TPA)	6800			5710		
No		Ref			Ref	
Yes		0.23 (0.21, 0.26)	<0.001		0.29 (0.25, 0.34)	<0.001

^1^ Model adjusted for follow-up time, baseline age, gender, education level, BMI, marital status, residency, income, drinking status, and smoking status.

**Table 4:** Association between FI and concurrent engagement in LePA based on longitudinal data from Waves 2 to 5 of CHARLS with results shown for both unadjusted and adjusted models.

Type of PA	FI					
	Unadjusted Model			Adjusted Model ^1^		
	*N*	OR (95% CI)	*p Value*	*N*	OR (95% CI)	*p Value*
Leisure Physical Activity (LePA)	6432			5248		
No		Ref			Ref	
Yes		0.56 (0.50, 0.64)	<0.001		0.59 (0.51, 0.68)	<0.001

^1^ Model adjusted for follow-up time, baseline age, gender, education level, BMI, marital status, residency, income, drinking status, and smoking status.

**Table 5:** Association between FI and different types of PA at both lagged time (one wave prior) and current time based on longitudinal data from Waves 1 to 5 of CHARLS with results shown for both unadjusted and adjusted models.

Type of PA	FI					
	Unadjusted Model			Adjusted Model ^1^		
	*N*	OR (95% CI)	*p Value*	*N*	OR (95% CI)	*p Value*
Current Light Physical Activity (LPA)	5271			4146		
No		Ref			Ref	
Yes		0.36 (0.31, 0.42)	<0.001		0.43 (0.35, 0.51)	<0.001
Previous (one-wave lagged) Light Physical Activity (LPA)						
No		Ref			Ref	
Yes		0.58 (0.49, 0.69)	<0.001		0.73 (0.60, 0.88)	0.001
Current Moderate Physical Activity (MPA)	5277			4152		
No		Ref			Ref	
Yes		0.37 (0.31, 0.43)	<0.001		0.43 (0.36, 0.52)	<0.001
Previous (one-wave lagged) Moderate Physical Activity (MPA)						
No		Ref			Ref	
Yes		0.58 (0.50, 0.67)	<0.001		0.66 (0.55, 0.78)	<0.001
Current Vigorous Physical Activity (VPA)	5275			4151		
No		Ref			Ref	
Yes		0.41 (0.34, 0.49)	<0.001		0.49 (0.39, 0.60)	<0.001
Previous (one-wave lagged) Vigorous Physical Activity (VPA)						
No		Ref			Ref	
Yes		0.58 (0.48, 0.69)	<0.001		0.78 (0.64, 0.96)	0.018
Current Total Physical Activity (TPA)	5239			4119		
No		Ref			Ref	
Yes		0.24 (0.20, 0.28)	<0.001		0.27 (0.22, 0.33)	<0.001
Previous (one-wave lagged) Total Physical Activity (TPA)						
No		Ref			Ref	
Yes		0.47 (0.40, 0.55)	<0.001		0.61 (0.50, 0.75)	<0.001

^1^ Model adjusted for follow-up time, baseline age, gender, education level, BMI, marital status, residency, income, drinking status, and smoking status.

**Table 6:** Association between FI and LePA at both lagged time (one wave prior) and current time based on longitudinal data from Waves 2 to 5 of CHARLS with results shown for both unadjusted and adjusted models.

Type of PA	FI					
	Unadjusted Model			Adjusted Model ^1^		
	*N*	OR (95% CI)	*p Value*	*N*	OR (95% CI)	*p Value*
Current Leisure Physical Activity (LePA)	4632			3678		
No		Ref			Ref	
Yes		0.56 (0.46, 0.67)	<0.001		0.61 (0.51, 0.74)	<0.001
Lagged Leisure Physical Activity (LePA)						
No		Ref			Ref	
Yes		0.75 (0.62, 0.90)	0.002		0.77 (0.63, 0.93)	0.007

^1^ Model adjusted for follow-up time, baseline age, gender, education level, BMI, marital status, residency, income, drinking status, and smoking status.

## Data Availability

The data used in this study were obtained from the publicly available China Health and Retirement Longitudinal Study (CHARLS) database, which is hosted by the National School of Development at Peking University. The CHARLS dataset is accessible to researchers through an application process to ensure compliance with privacy and ethical considerations. Researchers can request access to the data at http://charls.pku.edu.cn/en (accessed on 4 January 2025).

## Appendix A

**Table A1:** The 35 items used to construct the frailty index.

Domain	Item Number	Definition	Cut-Off Point
Diseases and Symptoms	1	Self-report general health status	Fair and Poor and Very poor = 1; Very good and Good = 0
	2	Self-reported physician diagnosed hypertension	Yes = 1, No = 0
	3	Self-reported physician diagnosed diabetes	Yes = 1, No = 0
	4	Self-reported physician diagnosed cancer	Yes = 1, No = 0
	5	Self-reported physician diagnosed chronic lung diseases	Yes = 1, No = 0
	6	Self-reported physician diagnosed heart problem	Yes = 1, No = 0
	7	Self-reported physician diagnosed stroke	Yes = 1, No = 0
	8	Self-reported physician diagnosed emotional, nervous, or psychiatric problems	Yes = 1, No = 0
	9	Self-reported physician diagnosed arthritis	Yes = 1, No = 0
	10	Self-reported physician diagnosed dyslipidemia	Yes = 1, No = 0
	11	Self-reported physician diagnosed liver disease	Yes = 1, No = 0
	12	Self-reported physician diagnosed kidney disease	Yes = 1, No = 0
	13	Self-reported physician diagnosed stomach or other digestive disease	Yes = 1, No = 0
	14	Self-reported physician diagnosed asthma	Yes = 1, No = 0
	15	Self-reported physician diagnosed memory-related disease	Yes = 1, No = 0
Disabilities	16	Some difficulty with dressing	Yes = 1, No = 0
	17	Some difficulty with bathing or showering	Yes = 1, No = 0
	18	Some difficulty with eating	Yes = 1, No = 0
	19	Some difficulty with getting into or out of bed	Yes = 1, No = 0
	20	Some difficulty with using the toilet	Yes = 1, No = 0
	21	Some difficulty with controlling urination and defecation	Yes = 1, No = 0
	22	Some difficulty with managing money	Yes = 1, No = 0
	23	Some difficulty with taking medications	Yes = 1, No = 0
	24	Some difficulty with shopping for groceries	Yes = 1, No = 0
	25	Some difficulty with preparing hot meals	Yes = 1, No = 0
	26	Some difficulty with household chores	Yes = 1, No = 0
Mobility	27	Some difficulty with walking 100 m	Yes = 1, No = 0
	28	Some difficulty with getting up from a chair	Yes = 1, No = 0
	29	Some difficulty with climbing several flights of stairs without resting	Yes = 1, No = 0
	30	Some difficulty with stooping, kneeling, or crouching	Yes = 1, No = 0
	31	Some difficulty with reaching or extending arms	Yes = 1, No = 0
	32	Some difficulty with lifting or carrying weights over 10 jin	Yes = 1, No = 0
	33	Some difficulty with picking up a small coin	Yes = 1, No = 0
Depression	34	CESD-10 questionnaire	CESD-10 > 10 = 1, CESD-10 ≤ 10 = 0
Cognition	35	(Visual-spatial ability + episodic memory + mental status)/21	Continuous variable ranging from 0 to 1

Depression was assessed using the Center for Epidemiologic Studies Depression Scale (CESD-10) in CHARLS, with total scores ranging from 0 to 30. Higher scores indicate more severe depressive symptoms.

Cognitive function was assessed across three domains: visual-spatial ability, episodic memory, and mental status. These were measured using the pentagon figure-drawing test, word recall tasks, and the Telephone Interview for Cognitive Status (TICS-10), respectively. The drawing test was scored as 1 for a correct drawing and 0 otherwise. Episodic memory was evaluated through the average of immediate and delayed recall of 10 unique Chinese nouns, with scores ranging from 0 to 10. Mental status was measured using TICS-10, which included 10 items such as date orientation and serial subtraction (subtracting 7 from 100 up to four times), with total scores ranging from 0 to 10.
